# A Simulation Model for the Non-Electrogenic Uniport Carrier-Assisted Transport of Ions across Lipid Membranes

**DOI:** 10.3390/membranes12030292

**Published:** 2022-03-03

**Authors:** Mariano Andrea Scorciapino, Giacomo Picci, Roberto Quesada, Vito Lippolis, Claudia Caltagirone

**Affiliations:** 1 Department of Chemical and Geological Sciences, Cittadella Universitaria di Monserrato, University of Cagliari–S.P. 8 km 0,700, I-09042 Monserrato, Italy; gpicci@unica.it (G.P.); lippolis@unica.it (V.L.); ccaltagirone@unica.it (C.C.); 2 Departmento de Quìmica, Universidad de Burgos, 09001 Burgos, Spain; rquesada@ubu.es

**Keywords:** anion receptors, anion recognition, chloride transport, dipicolineamide, lipid vesicles, squaramide, supramolecular medicinal chemistry, synthetic transporters, variable time-step, water to lipid partition

## Abstract

Impressive work has been completed in recent decades on the transmembrane anion transport capability of small synthetic transporters from many different structural classes. However, very few predicting models have been proposed for the fast screening of compound libraries before spending time and resources on the laboratory bench for their synthesis. In this work, a new approach is presented which aims at describing the transport process by taking all the steps into explicit consideration, and includes all possible experiment-derived parameters. The algorithm is able to simulate the macroscopic experiments performed with lipid vesicles to assess the ion-transport ability of the synthetic transporters following a non-electrogenic uniport mechanism. While keeping calculation time affordable, the final goal is the curve-fitting of real experimental data—so, to obtain both an analysis and a predictive tool. The role and the relative weight of the different parameters is discussed and the agreement with the literature is shown by using the simulations of a virtual benchmark case. The fitting of real experimental curves is also shown for two transporters of different structural type.

## 1. Introduction

Since the pioneering work on chelating Lewis acids [1] and ammonium-based cryptand halide receptors [2] in the late 1960s, the anion receptor chemistry has become one of the most popular topics in the realm of supramolecular chemistry [3,4,5,6,7,8]. Impressive work has been completed, covering different aspects such as anion recognition, sensing, extraction, crystal engineering, self-assembling, catalysis, and transmembrane anion transport. The latter has lately attracted considerable attention, as transmembrane anion transport is crucial to several biological processes [9,10,11,12,13,14,15]. Failure in maintaining chloride homeostasis in cells is related to various diseases [16] such as cystic fibrosis, in which a malfunctioning of the cystic fibrosis transmembrane conductance regulator (CFTR) gene causes defective chloride transport [17]. The development of artificial chloride transmembrane transporters, as substitutes for such defective anion channels, has been tackled during the last two decades [18].

When designing an artificial chloride transporter, with the aim of eventually testing it in vivo and using it as a drug for patience treatment, the optimization of ADME (absorption, diffusion, metabolism and excretion) properties is clearly crucial [19]. This can be achieved by using small molecules (low molecular mass) properly functionalized to obtain suitable logP values (i.e., the log of the octanol–water partitioning coefficient). The interaction between the chloride ion and the transporter is often established via the formation of hydrogen bonds. Indeed, ureas and thioureas, as well as squaramides and sulfonamides, have been proven to be potent chloride transmembrane transporters. When studying the potential ability of a class of receptors as chloride transmembrane artificial transporters, after evaluating important parameters such as the chloride affinity in solution (often in organic solvents such as DMSO or chloroform) and the lipophilicity, a series of transport assays with ad-hoc-prepared vesicles dispersions, such as the Cl^−^/NO_3_^−^ antiport assay, are conducted to elucidate and compare the transport ability of each transporter. This approach is time- and money-consuming. Very often, only a few receptors from a vast library show good transmembrane transport activity. In order to save time and to reduce costs, the development of a reliable and simple numerical model, and an efficient computer-based method able to predict the transport abilities on the basis of the physical and chemical parameters of the species of interest, would be highly relevant to the scientific community.

Very few predicting models have been proposed in the literature so far. In 2013, Berezin proposed an interesting application of the Goldman–Hodgkin–Katz (GHK) theory and kinetic modelling to study the function of the anion transporters in liposomes [20,21]. However, certain assumptions, which have the advantage of keeping the approach simple and fast, represent a reduction in flexibility when different scenarios need to be considered and the relative weight of the different physico-chemical parameters need to be unveiled. For instance, to assume a high partition for the transporter from the water to the lipid phase is not always adequate, since many efficient synthetic transporters do exist with a relatively low logP [9,22]. As another example, diffusivity of all the species of interest should be taken into explicit account, especially when the net charge of the carrier and the chloro-complex is different, in order to have a flexible model where either the carrier or the complex diffusion through the lipid membrane can be the rate limiting step, but that is also able to describe intermediate situations.

To take the available experimental data into account is extremely important and, in the same year, quantitative structure–activity relationship (QSAR) analysis on a family of thioureas highlighted that the transport activity mostly depends on the partitioning of the transporter into the membrane and, to a less but significant extent, on the diffusion of the carrier through the membrane and its anion-binding ability [23]. QSAR studies seek the development of mathematical models to relate descriptors, which can be both experimental physico-chemical properties and theoretically calculated parameters from sets of molecules, with activities or properties, in order to predict the response of new chemicals [24]. Although these models have proven to be potentially useful to predict the transmembrane chloride transport activity of artificial receptors, they are based on very few parameters, whose relative weights and, especially their inter-relationship, are impossible to be clearly deciphered. On the other hand, molecular dynamics simulations and DFT-level calculations have been successfully applied to several synthetic transporters and proven to be extremely useful to separately investigate different steps of the overall transport process at atomic-level detail [25,26,27,28], such as anion-binding or the carrier/complex migration through the lipids. However, such a level of accuracy does not come without a cost. The simulation of the experimental transport curves with such methods is not practically feasible, due to the current limitations of computers performance that restrict the investigations both on a size and time scale.

In this work, we present a different approach, which aims at describing the overall transport process by taking all the relevant steps into explicit consideration, and by including all the possible experiment-derived parameters, while keeping calculation time affordable with the clear goal of fitting real experimental data. We recently proposed an embryonal numerical model to fit and analyze the experimental data of the chloride transporter 3,4-bis((1H-indol-7-yl)amino)cyclobut-3-ene-1,2-dione [22]. We discuss here the development of that idea in detail. We quantitatively describe the partition of the carrier and the complex from the water to the lipid phase by taking into account the macroscopic quantities characterizing the vesicles dispersion used during the experiments. We find the equations to describe all the chemical equilibria pertaining to the model of interest and we solve them either analytically or numerically to calculate the concentration of all the species involved. We apply Fick’s law to the diffusion of both the carrier and the complex through the lipid membrane, and we iteratively execute these calculations at regular time-steps, so as to simulate the macroscopic experiments and reconstruct the curves of chloride release. We finally apply a Monte Carlo approach to perform the curve-fitting of real data. In the present work, we focused on the simplest mechanism, the uniport, which involves the carrier-mediated transport of a single type of ion. We suggest further development towards more complex and general mechanisms, although several improvements have already been put forward.

## 2. Methods

**Figure 1:**
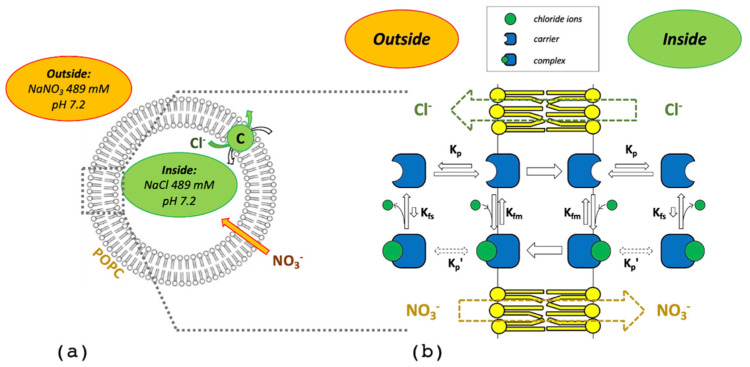
Non-electrogenic uniport model: (**a**) Typical experimental condition; (**b**) the four reversible equilibria considered in the model on both sides of the lipid membrane, together with the labels of the corresponding equilibrium constants: K_p_, the free carrier partition between the water and the lipid phase; K_fm_, the complex formation in the lipid phase; K_fs_, the complex formation in the water phase; K′_p_, the complex partition between the water and the lipid phase. The dashed–green and orange arrows indicate the net flux of chloride and nitrate ions, respectively.

In this work, we focused on the simplest carrier-assisted transport mechanism of ions across artificial lipid membranes, i.e., the non-electrogenic uniport. We selected, as an experimental benchmark, the case of the squaramide 3,4-bis((1H-indol-7-yl)amino)cyclobut-3-ene-1,2-dione carrier, recently published [22], and the chloride anion. The typical experimental conditions are schematically illustrated in Figure 1. A dispersion of 1-palmitoyl-2-oleoyl-sn-glycero-3-phosphocholine (POPC) vesicles with an average diameter of 200 nm was prepared. The extra-vesicular (EV) solution contained 489 mM NaNO_3_ and 10 mM phosphate buffer at pH 7.2. The intra-vesicular (IV) solution contained 489 mM NaCl and the same buffer at the same pH. Then, a given amount of carrier, typically reported as %_mol_ with respect to total lipid, was added to the external solution to initiate the transport process. The latter was monitored over time by a chloride-specific electrode in the EV solution under continuous gentle stirring. The carrier had zero net charge and a marked affinity for the lipid membrane, as indicated by the logP value of 2.83 [29,30], where P was the partition coefficient. According to the host–guest equilibrium H+G⇄HG, where the host H is the carrier and the guest G is the chloride ion, the carrier had a good affinity for chloride ions in a low polar environment, as indicated by the formation equilibrium constant of $1.2\times 10^{3}\;M^{-1}$ which was determined through ^1^H-NMR in DMSO/0.5% water [22]. However, carrier affinity for chloride dramatically dropped when the amount of water increased, with an observed formation equilibrium constant of $1.1\times 10^{2}\;M^{-1}$ and $2.1\times 10^{1}\;M^{-1}$ in DMSO/10% water and DMSO/25% water, respectively. On the other hand, carrier affinity for nitrate ions is negligible [22]. Thus, for the sake of simplicity, in this work, nitrate ions were considered not as the rate limiting step of the transport process but to cross the POPC bilayer (either by direct or carrier-assisted diffusion) keeping the charge across the membrane balanced. It should be noted that such a chloride/nitrate exchange mechanism has been described for both the carriers considered in the following (L1 and L2 in Section 3.9) and it is consistent with the larger lipophilicity of nitrate when compared to chloride.

### 2.1. Chemical Equilibria

On both sides of the membrane, four reversible equilibria were considered (Figure 1b). The chemical equation and the corresponding equilibrium constant for the complex formation in the lipid (Equation (1)) and in the water phase (Equation (2)) were:

(1) $$H_{m}+G⇄{\mathrm{HG}}_{m};\;K_{\mathrm{fm}}=\frac{{\left[\mathrm{HG}\right]}_{m}}{{\left[H\right]}_{m}\left[G\right]}$$

(2) $$H_{s}+G⇄{\mathrm{HG}}_{s};\;K_{\mathrm{fs}\;}=\frac{{\left[\mathrm{HG}\right]}_{s}}{{\left[H\right]}_{s}\left[G\right]}$$

where m and s subscript indicate the membrane and the solution, respectively. H (host) is the carrier, G (guest) is the chloride ion, and HG is the complex. K_fm_ is the formation equilibrium constant in the membrane. K_fs_ is the formation equilibrium constant in solution. Square brackets are used to indicate molar concentration.

The above-mentioned experimental data of ^1^H-NMR titration in DMSO-d_6_ with increasing amounts of water could be fitted with the following exponential function:

(3) $$K_{f}=a\cdot e^{-b\cdot w\%},\;\mathrm{with}\;a=1480\;\pm \;26\;\mathrm{and}\;b=0.26\;\pm \;0.02,$$

where w% is the water amount in volume percentage. Thus, the value of a (in units of M^−1^) was taken as the K_fm_, while the K_fs_ was obtained by setting w% at 100. The latter resulted to be as low as 8.34 × 10^−9^ M^−1^. Although this is negligible, we decided to include Equation (2) in our model anyway, in order to keep it general.

The association among molecules, and among molecules and ions, is typically described in terms of chemical equilibria according to a specific stoichiometry. Solvent concentration can be considered constant and included in the equilibrium constant. For the same reason, solvent is omitted from chemical equations. While this is quite valid for host–guest association in diluted solution, or even between the guest and the membrane-bound host, it cannot be applied to partition equilibria of species between the water and the lipid phase [31]. In this case, water and lipid cannot be omitted and must be explicitly considered. The chemical equation and the corresponding equilibrium constant for the partition between the water and the lipid phase of the free carrier (Equation (4)) and the complex (Equation (5)) are:

(4) $$H_{s}+L⇄H_{m}+W;\;K_{p}=\frac{{\left[H\right]}_{m}\left[W\right]}{{\left[H\right]}_{s}\left[L\right]}$$

(5) $${\mathrm{HG}}_{s}+L⇄{\mathrm{HG}}_{m}+W;\;{K^{\prime }}_{p}=\frac{{\left[\mathrm{HG}\right]}_{m}\left[W\right]}{{\left[\mathrm{HG}\right]}_{s}\left[L\right]}$$

where L and W indicate lipid and water molecules, respectively. K_p_ and K′_p_ are the thermodynamic partition equilibrium constants of the free carrier and the complex, respectively. The thermodynamic constant can be difficult to experimentally determine, so the partition coefficient P is typically used to compare the lipophilicity of different compounds [31]. This is a more convenient parameter which is usually determined as the ratio between the concentration of the species of interest in the octan-1-ol and in the water phase in a separatory funnel. Many databases, webservers and software products are also available to estimate the *p* value [29,32,33,34]. A suitable relation between the thermodynamic K_p_ and the empirical P is presented in the literature [35,36], allowing a proper and convenient description of the partition equilibrium:

(6) P=nmVLnsVW=KpγWγL

where n is the number of moles of the species of interest in the membrane or in the solution, V is the volume occupied by the lipid or the water phase, and γ is the molar volume of the water or the lipid molecules. This relation was successfully applied to describe the partition of peptides between the water solution and the lipid membrane of unilamellar vesicles [31,35,36,37] (as in the present case). Thus, by starting from Equation (4) and by using Equation (6), it is possible to write [H]_m_ as a function of [H]_s_ as:

(7) $${\left[H\right]}_{m}=\frac{K_{p}{\left[H\right]}_{s}\left[L\right]}{\left[W\right]}=\frac{{P\gamma }_{L}{\left[H\right]}_{s}\left[L\right]}{\gamma_{W}\left[W\right]}{=P\gamma }_{L}{\left[H\right]}_{s}\left[L\right]$$

where the last simplification is possible by noting that $V_{W}\approx V_{\mathrm{tot}}$. Then, by starting from Equation (1) and by substituting Equation (7), it is possible to write [HG]_m_ as a function of [H]_s_ as:

(8) $${\left[\mathrm{HG}\right]}_{m}{=K}_{\mathrm{fm}}{\left[H\right]}_{m}\left[G\right]{=P\gamma }_{L}{\left[H\right]}_{s}\left[L\right]K_{\mathrm{fm}}\left[G\right]$$

Finally, Equation (2) is simply rearranged as:

(9) $${\left[\mathrm{HG}\right]}_{s}{=K}_{\mathrm{fs}}{\left[H\right]}_{s}\left[G\right]$$

On the right-hand side of Equations (8) and (9), the only other variable is [G], which can be expressed as function of [H]_s_ as:

(10) $$\left[G\right]={\left[G\right]}_{\mathrm{tot}}-{\left[\mathrm{HG}\right]}_{m}-{\left[\mathrm{HG}\right]}_{s}={\left[G\right]}_{\mathrm{tot}}{-P\gamma }_{L}{\left[H\right]}_{s}\left[L\right]K_{\mathrm{fm}}\left[G\right]{-K}_{\mathrm{fs}}{\left[H\right]}_{s}\left[G\right]$$

by substituting Equations (8) and (9). Then, Equation (10) can be rearranged as:

(11) [G]=[G]tot(1+PγL[H]s[L]Kfm+Kfs[H]s)

Similarly, we can write an expression for the total carrier concentration:

(12) $${\left[H\right]}_{\mathrm{tot}}={\left[H\right]}_{s}+{\left[\mathrm{HG}\right]}_{s}+{\left[H\right]}_{m}+{\left[\mathrm{HG}\right]}_{m}$$

which, after substitution with Equations (7)–(9) and (11), can be rearranged as:

(13) $$\begin{array}{l} {\left[H\right]}_{s}{}^{2}\left(P^{2}\gamma_{L}{}^{2}{\left[L\right]}^{2}K_{\mathrm{fm}}{+P\gamma }_{L}\left[L\right]K_{\mathrm{fm}}{+P\gamma }_{L}\left[L\right]K_{\mathrm{fs}}{+K}_{\mathrm{fs}}\right)+{\left[H\right]}_{s}({1+K}_{\mathrm{fs}}{\left[G\right]}_{\mathrm{tot}}{+P\gamma }_{L}\left[L\right]+\\ {+P\gamma }_{L}\left[L\right]K_{\mathrm{fm}}{\left[G\right]}_{\mathrm{tot}}{-P\gamma }_{L}\left[L\right]K_{\mathrm{fm}}{\left[H\right]}_{\mathrm{tot}}{-K}_{\mathrm{fs}}{\left[H\right]}_{\mathrm{tot}})-{\left[H\right]}_{\mathrm{tot}}=0 \end{array}$$

This is a second-order equation that can be analytically solved to find [H]_s_ at equilibrium for the given P, total lipid concentration [L], K_fm_, K_fs_, total carrier and chloride concentration. The molar volume of the employed lipids is needed. In the case of the POPC considered in this work, the value 0.762 L mol^−1^ was applied [38]. After [H]_s_ is calculated, it can be substituted back into Equation (11), and then in Equations (7)–(9), to calculate [G], [H]_m_, [HG]_m_ and [HG]_s_, respectively.

From a practical viewpoint, it is important to note that the complex partition constant K′_p_, is not needed and, thus, is not to be experimentally determined (it is dashed in Figure 1). The K′_p_ results to be determined by the other 3 equilibrium constants as:

(14) $${K^{\prime }}_{p}{=K}_{p}\frac{K_{\mathrm{fm}}}{K_{\mathrm{fs}}}$$

It is straightforward to move from concentration to the number of moles by multiplying with the volume. However, in the present context, we need to distinguish the EV from the IV solution and to resolve the equilibrium Equations separately for the two compartments.

### 2.2. Vesicles Description

In order to determine the EV volume and IV volume, V_out_ and V_in_, respectively, we applied a numerical approach already reported in the literature [20,37,39].

Briefly, unilamellar spherical vesicles with identical diameter, *d*, were assumed. The diameter was taken as 200 nm from the pore size of the polycarbonate filter used during the extrusion stage of vesicles preparation [22]. The number of lipid molecules in one vesicle was calculated (by neglecting the difference between the area of the external and internal leaflet of the lipid bilayer) by simply doubling the vesicle area and then multiplying the result with the lipid surface density, $\sigma_{L}$. The value of 1.3 nm^−2^ was determined through surface tension measurements of the POPC monolayer at the air/water interface [37] and it was in agreement with the value reported by other authors [40,41]. Then, at a given total lipid concentration, the number of vesicles present (n_v_) in the whole sample, the total (single side) vesicles’ area (A_v_) and the total internal volume (V_in_) could be calculated:

(15) nv=[L]VtotNA8π(d2)2σL

(16) $$A_{v}=\frac{\left[L\right]V_{\mathrm{tot}}N_{A}}{{2\sigma }_{L}}$$

(17) $$V_{\mathrm{in}}=\frac{\left[L\right]V_{\mathrm{tot}}N_{A}d}{{12\sigma }_{L}}\cdot 1000$$

where N_A_ is the Avogadro’s number and the 1000 multiplication in Equation (17) is needed to change from m^3^ to L units. V_out_ is obtained by difference with V_tot_.

In a successive version of the algorithm, the difference between the area of the external and internal leaflet of the lipid bilayer was considered. The value of d2 was kept as the external radius, while d2−w was used as the internal diameter. Accordingly, the external and internal areas per vesicle were different, resulting in a corrected value for n_v_ and, in turn, for V_in_ (but not for V_out_). Instead of a single side A_v_ used for both the compartments, two distinct values of total area were used, A_v,out_ and A_v,in_, for the EV and the IV compartment, respectively.

### 2.3. Transport Stage and Iteration

After concentrations and corresponding moles were obtained on both sides of the membrane, flux density was calculated for both the membrane-bound carrier (J) and complex (J’) through Fick’s law:

(18) $$J=-\frac{D}{w}\left({\left[H\right]}_{m,\mathrm{out}}-{\left[H\right]}_{m,\mathrm{in}}\right)\cdot 1000$$

(19) $$J’=-\frac{D_{c}}{w}\left({\left[\mathrm{HG}\right]}_{m,\mathrm{out}}-{\left[\mathrm{HG}\right]}_{m,\mathrm{in}}\right)\cdot 1000$$

where w is the membrane hydrophobic thickness, D and D_c_ are the carrier and the complex diffusion coefficient through the membrane, respectively, and multiplication by 1000 is needed to change from mol L^−1^ to mol m^−3^ and, thus, to have the flux density expressed in mol m^−2^ s^−1^ units. It is important to note that in the present model, D and D_c_ are explicitly and separately taken into account. The value of D and D_c_, at least in principle, might be estimated through the Stokes–Einstein relation. However, an isotropic medium and an almost spherical shape are assumed by the latter, which might lead to very large errors [42]. In addition, when the carrier and the complex are compared, although size can sometimes be considered comparable, the conformational plasticity as well as the charge usually change. These have a tremendous impact on permeability, with a difference from neutral to charged species of orders of magnitude [42,43,44]. In the present work, selected values of D and D_c_ were applied just to investigate their weight on the overall transport process and, in the case of experimental data curve-fitting (see below), we used them as the fitting parameters. As far as the hydrophobic thickness of the POPC bilayer is concerned, the value of $2.85\times 10^{-9}\;m$ was considered [43,45].

Finally, the flux of both the membrane-bound carrier and complex from one side of the membrane to the other one can be easily calculated by multiplying the flux density with both A_v_ and a suitable time-step, t_s_. The new values of the moles for both the membrane-bound carrier and the membrane-bound complex are summed to the carrier and the complex in solution in order to obtain an updated value of total host and total guest on either side of the membrane. Basically, in the present model, equilibria were assumed to be much faster than the permeation through the membrane, so that as soon as species amount was updated, all the above-mentioned equilibria were instantaneously reestablished. This means, new concentration gradients were evaluated, thus, creating new flux densities. Again, the latter were multiplied by A_v_ and t_s_ to obtain the mole flux during a further time-step, and the iteration was repeated up to the total simulation time (a typical experiment lasted for 300 s).

At each time-step, the algorithm also checks for the total amount of host and guest in the entire system. Moreover, vesicles surface coverage is monitored, by providing an estimated value for the area occupied by a single molecule of carrier and complex. Guest release in the EV solution is calculated as ratio between [G]_s,out_ and [G]_tot_. The initial release rate, k_ini_, is obtained from the first point of the output files divided by the corresponding simulated time (typically 5 s). The half maximal effective concentration, EC_50_ (i.e., the carrier concentration needed to reach 50% of maximal release at 270 s [9,23]) was directly obtained by changing the carrier concentration and by checking the release at 270 s.

### 2.4. From Fixed to Variable Time-Step

The time-step has to be suitably short, in order to avoid simulation instability and physically unacceptable numbers. If the time-step is too long, mole flux will be too large, leading to a considerable amount of the species leaving one of the compartments and possibly resulting in negative concentrations. On the other hand, if the time-step is too short, the simulation will certainly be stable, but it will take too long to be completed.

As a rule-of-thumb, the time-step should be set in order to have a reduction in the species leaving a given compartment up to a maximum of 10%. In the typical conditions pertaining to the present work, 1 μs was found to be a suitable choice. For the sake of completeness, it has to be mentioned that also time-steps as short as 1 ns were checked, leading to identical results.

In cases analogous to the selected benchmark, the maximum density flux was observed at the beginning of the simulation, when the concentration of the carrier outside was at the maximum and inside the vesicles was zero, and chloride concentration inside the vesicles was at the maximum and zero outside. Concentration gradients fade along the simulation, and so does the flux density, accordingly, which means that a longer time-step could be progressively applied without affecting simulation results, shortening the time for simulation completion significantly. Thus, in a successive version of the algorithm, the fixed pre-selected time-step was replaced by a variable time-step approach.

For every given number of the time-steps (usually selected to be equal to the experimental dwell time of 5 s), the maximum flux density is selected (absolute value) to check whether the time-step can be increased. At each iteration, on each membrane leaflet,

(20) $$n_{i,t}{=n}_{i,\left(t-1\right)}\;\pm \;J\cdot A_{v}\cdot t_{s}$$

where n_i,t_ and n_i,(t−1)_ are the number of moles of the i-th species at time t and t−1 (in t_s_ units), respectively. The sign of J (or J’) depends on the sign of the concentration gradients (Equations (18) and (19)). We need to ensure that the right-hand side of Equation (20) is >0. As mentioned, a suitable condition is that reduction in the number of moles is 10% at the maximum, which is:

(21) $$\left|J\right|A_{v}t_{s}\leq \;0{.1n}_{i,\left(t-1\right)}$$

Thus, the following condition must be met:

(22) $$t_{s}\leq \frac{n_{i,\left(t-1\right)}}{10\left|J\right|A_{v}}$$

Close to final equilibrium, J→0, so it is mandatory to set a limit on the maximum t_s_, which was typically selected as 1 ms.

### 2.5. Reversible Aggregation: From Analytical to Numerical Roots

**Figure 2:**
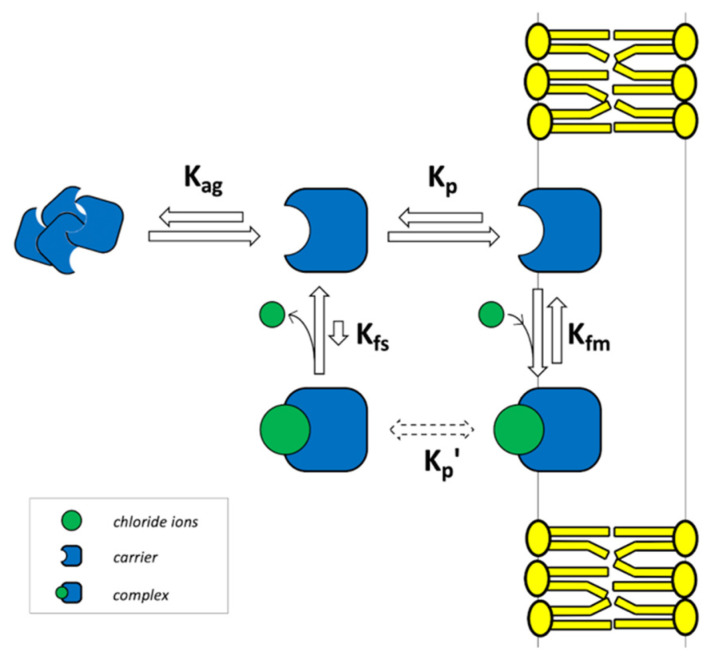
Non-electrogenic uniport model including carrier aggregation. The four reversible equilibria considered in the model on both sides of the lipid membrane, together with the labels of the corresponding equilibrium constants: K_p_, the free carrier partition between the water and the lipid phase; K_fm_, the complex formation in the lipid phase; K_fs_, the complex formation in the water phase; K′_p_, the complex partition between the water and the lipid phase; K_ag_, the carrier aggregation in solution.

The model shown in Figure 1 was extended to take into account the possible aggregation of the carrier in solution. The modified scheme is shown in Figure 2.

Reversible aggregation is expressed by the following chemical equation:

(23) $$\mathrm{zH}⇄H_{z};\;K_{\mathrm{ag}}=\frac{\left[H_{z}\right]}{{\left[H\right]}^{z}}$$

which provides a microscopic description of the carrier aggregation by taking stoichiometry into explicit account [46] where z is the aggregation number, i.e., the number of monomers present in each aggregate. By following the same procedure as in Section 2.1, and noting that now, instead of Equation (12), we have:

(24) $${\left[H\right]}_{\mathrm{tot}}={\left[H\right]}_{s}+{\left[\mathrm{HG}\right]}_{s}+{\left[H\right]}_{m}+{\left[\mathrm{HG}\right]}_{m}+z\left[H_{z}\right]$$

the final equation for [H]_s_ is not of the second but of the (z + 1)-th order:

(25) $$\begin{array}{l} {\left[H\right]}_{s}{}^{z+1}\left({\mathrm{zK}}_{\mathrm{ag}}K_{\mathrm{fs}}{+\mathrm{zK}}_{\mathrm{ag}}K_{\mathrm{fm}}{P\gamma }_{L}\left[L\right]\right)+{\left[H\right]}_{s}{}^{z}\left(z\cdot K_{\mathrm{ag}}\right)+{\left[H\right]}_{s}{}^{2}{(P}^{2}\gamma_{L}{}^{2}{\left[L\right]}^{2}K_{\mathrm{fm}}\\ {+P\gamma }_{L}\left[L\right]K_{\mathrm{fm}}{+P\gamma }_{L}\left[L\right]K_{\mathrm{fs}}{+K}_{\mathrm{fs}})+{\left[H\right]}_{s}{(1+K}_{\mathrm{fs}}{\left[G\right]}_{\mathrm{tot}}{+P\gamma }_{L}\left[L\right]{+P\gamma }_{L}\left[L\right]K_{\mathrm{fm}}{\left[G\right]}_{\mathrm{tot}}\\ {-P\gamma }_{L}\left[L\right]K_{\mathrm{fm}}{\left[H\right]}_{\mathrm{tot}}-K_{\mathrm{fs}}{\left[H\right]}_{\mathrm{tot}})-{\left[H\right]}_{\mathrm{tot}}=0 \end{array}$$

The analytical solution for equations of orders larger than 4 are not available as far as we are aware. In addition, to make the algorithm general and ready for even more complex models, we decided to move from the analytical to the numerical root search. We opted for the well-known Newton–Raphson method. The initial guess of the root is the total concentration of the carrier in the given compartment. The algorithm will find the closest positive real root lower than the initial guess, which is the desired one.

### 2.6. Curve-Fitting of Experimental Data

A Monte Carlo scheme was used to minimize the sum of square differences between the experimental and the calculated data points by using the diffusion coefficient of both the carrier and the complex through the lipid bilayer as fitting parameters. The algorithm can be easily extended to a larger number of fitting parameters.

At each iteration, one of the fitting parameters is randomly selected and randomly varied between ±var% of its current value (typically 10%), and the entire simulation from 0 to 300 s is performed from scratch. Only the moves resulting in a decrease in the sum of square differences between the experimental and the calculated data points are accepted, otherwise the last move is rejected. All the datasets pertaining to different carrier concentrations are fitted independently. The random number generator is automatically seeded with respect to the system clock.

Such a minimization method may suffer from the starting conditions and may converge rather slowly. Thus, by taking advantage of the speed gained through the variable time-step approach (Section 2.4), a specific module was inserted in the algorithm to search for good starting values of the fitting parameters. Basically, by deciding a maximum and a minimum value for the parameters, as well as a sampling interval, a screening is performed by comparing the sum of square difference from the experimental data of the simulated curves. The initial screening should be carefully set, since the number of simulations to be performed is given by $\underset{i}{\prod }N_{i}$, where N is the number of different values to be screened for each of the i fitting parameters.

## 3. Results and Discussion

All the numerical tests described here were performed by changing one parameter at a time with respect to a reference set, which is reported in Appendix A, unless differently indicated. Values for these parameters were chosen on the basis of our own experimental data, from the literature or estimated as explained in the Methods. In particular, given all the other parameters, the diffusion coefficient of the free carrier (D) and the complex (D_c_) were set identical, and the value was selected in order to reach the final equilibrium during the last 50 s of the simulation.

### 3.1. Simulation Output

**Figure 3:**
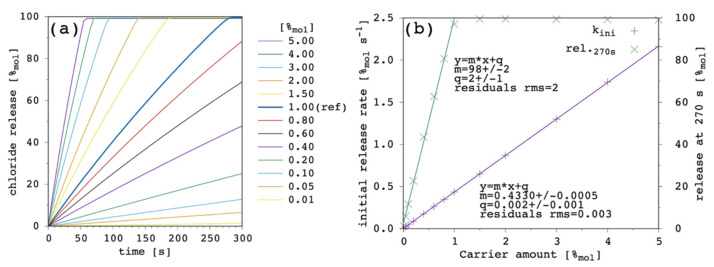
Simulated chloride transport according to the reference parameter set (Appendix A) by changing the amount of carrier: (**a**) release curves; (**b**) the corresponding initial rate and the release at 270 s are shown as a function of the carrier amount. The least-square linear curve fittings are also shown.

The most important result of the presented simulation model is certainly the release curve, i.e., the amount of chloride ions released in the EV solution as a function of time. This is the curve to be compared with the typical experimental data from release assays and which is analyzed to characterize and compare the transporter efficacy in terms of the initial release rate, k_ini_ [9,23]. One series of release curves, obtained by varying the carrier amount, are further analyzed to determine the carrier concentration facilitating 50% efflux of encapsulated chloride in the chosen timeframe (270 s), EC_50_ [9,23]. Figure 3 shows the effect of changing the carrier concentration within the present model.

Transport rate increases with increasing the amount of carrier in the system as one can expect. The initial release rate, k_ini_, linearly increases. Release curves at all the tested carrier concentrations showed an almost linear trend up to the final equilibrium (or to the end of the simulation), corresponding to virtually 100%_mol_ of chloride ions being released to the EV solution. For each of the simulated release curves, the ordinate at 270 s was extracted and reported vs. the carrier amount in Figure 3b. Moreover, the release at 270 s showed a linear dependence on the carrier concentration, from which an EC_50_ of 0.49%_mol_ was obtained. Such a linear trend of the release at 270 s vs. the carrier amount is hardly observed in real systems, for which an hyperbolic/sigmoid trend is typical and data can be fitted with the Hill’s model [9,23]. This point will be discussed later (Section 3.6) but, nevertheless, the following results and considerations are shown to be consistent with the literature.

**Figure 4:**
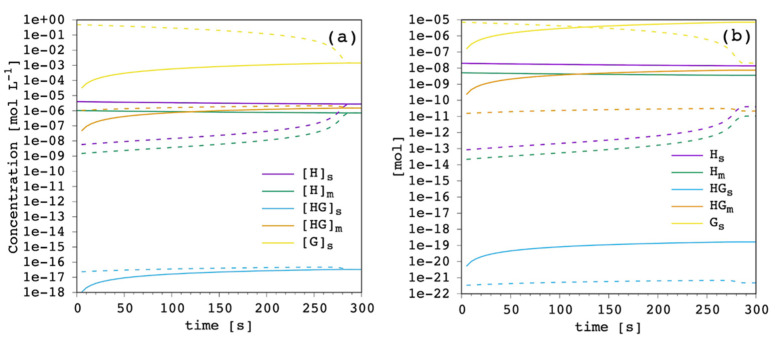
Simulated chloride transport according to the reference parameter set (Appendix A): (**a**) concentration of all the species in both the extra-vesicular (EV) solution (solid lines) and the intra-vesicular (IV) solution (dashed lines); (**b**) the corresponding amount of moles.

For each simulation, the concentration of each species and the moles can be monitored as a function of time in both compartments, the EV and IV solution. The results for the reference case with carrier amount equal to 1%_mol_ to total lipid are shown in Figure 4.

The free carrier in the EV solution (purple in Figure 4) showed an almost constant concentration throughout the simulation, meaning that it works as an effective reservoir. The concentration of free carrier in the IV solution slowly increased along the simulation up to the equilibrium when the concentration in the IV solution reached that of the EV compartment. The trend of the free membrane-bound carrier (green in Figure 4) was identical, both in the EV and IV compartments, to the free unbound carrier. Their molar ratio was constant, simply reflecting the value of the carrier’s partition coefficient. As it was expected from the extremely low value of the complex formation equilibrium constant in solution, the concentration of this species (cyan in Figure 4) was quite negligible throughout the simulation, both in the EV and the IV solution. However, it is important to stress that this equilibrium is explicitly included in our model, making it flexible and applicable to a wider range of carriers. The membrane-bound complex showed the same trend, although with significant concentration, reflecting the complex partition coefficient and the large formation constant in the membrane-bound state. The trend of the free chloride ions was coherently the same.

At the beginning of the simulation, chloride concentration was maximum in the IV solution, shifting all the equilibria towards complex formation. Progressively, chloride ions were released in the EV solution thanks to the extremely low complex formation constant in solution and the almost 350 times larger EV volume compared to the IV one. Thus, chloride ion concentration in the IV solution decreased along the simulation, and correspondingly increased in the EV solution. At the equilibrium, concentration in the EV and IV solution was the same for each species, with the molar ratio simply reflecting the corresponding equilibrium constants. Finally, Figure 4b shows how, besides free chloride ions, the most abundant species are always the ones in the EV solution (but the complex in solution), while the amount of all the species in the IV solution is less by orders of magnitude. This is directly related to the EV/IV volume ratio.

### 3.2. Carrier’s Partition Coefficient

**Figure 5:**
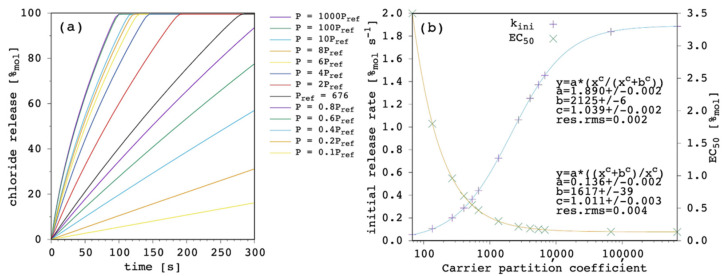
Simulated chloride transport according to the reference parameter set (Appendix A) by changing the carrier’s partition coefficient: (**a**) release curves; (**b**) the corresponding initial rate and the EC_50_ are shown as a function of the carrier’s partition coefficient. The least-square curve fittings are also shown.

A larger partition coefficient of the carrier, P, means that the latter is more lipophilic (or less hydrophilic). As a result, the amount of membrane bound species increases, both for the free carrier and the complex. This should, in turn, increase the concentration gradients across the lipid bilayer, providing a faster chloride release overall. Figure 5 shows the results of our simulations.

The effect of changing P is large. At high values of P, release curves also show a slight deviation from linearity with small positive curvature. The k_ini_ increases according to a hyperbolic curve as a function of P and EC_50_ decreases according to a reciprocal hyperbolic curve.

Membrane permeability to a given species is not only dependent on the diffusivity of the species through the membrane components but also on its lipid/water partition coefficient [42,44,47]. In turn, the net flux of the species across the membrane depends on the partition, in addition to the hydrophobic thickness of the latter. Although the importance of carrier partition on transport efficacy has been widely recognized [9,23,43,48], some models do not take it into explicit account and/or focus the application to cases with very high partition, so that free carrier in solution can be neglected [20,21]. An extensive Quantitative Structure–Activity Relationship (QSAR) investigation performed on a large dataset from the literature [23] clearly showed the important weight of the partition. The proposed QSAR models are the linear combination of four terms: the first term describes lipophilicity and has a positive coefficient; the second term describes anion-binding affinity and has a positive coefficient as well; the third term describes the molecular size and has a negative coefficient; the fourth term is a negative constant. Although coefficients were obtained using the absolute value of the observables of interest and cannot be compared to each other to evaluate the relative weight of lipophilicity, anion binding and molecular size, the authors also proposed a scaled analysis and concluded that “variation in anion transport ability […] is dominated by lipophilicity with smaller, yet significant, contributions from anion binding and diffusion” [23].

The present model does not only agree with the literature in predicting the increase in transport efficacy with increasing P [49], but it takes the latter into explicit account. This makes the quantitative direct estimation of the weight of P accessible by selecting the binding affinity and the diffusion coefficient of interest. In addition, the present model reveals that a saturation is finally reached, as shown in Figure 5b. This is reasonable since, in fact, the amount of the membrane-bound species increases with increasing P and so do the net fluxes across the membrane but, eventually, the diffusion through the membrane itself becomes rate limiting, and no further significant improvement is observed for transport efficacy (this point will be shown and discussed later in further details; Section 3.6). This has a very practical consequence because in some instances, i.e., depending on the other parameters, the increase in carrier lipophilicity might exhibit only moderate advantages, while significantly increasing the risk of gaining aggregation and solubility problems. It should be noted, in fact, that experimental evidence generally points at optimal transport activity for a P range, while a decrease in activity is found at lower and, more interestingly, at larger *p* values [49], suggesting that transport efficacy can diminish as a consequence of the derived aggregation and solubility problems of the carrier.

### 3.3. Complex Formation Equilibrium Constant in the Membrane

**Figure 6:**
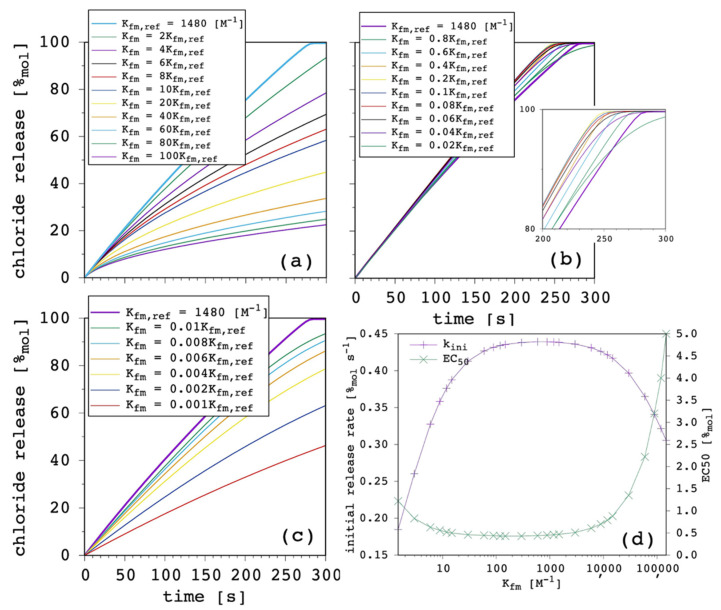
Simulated chloride transport according to the reference parameter set (Appendix A) by changing the complex formation equilibrium constant in the membrane: release curves with K_fm_ varying from (**a**) 1× to 100× the reference K_fm_; (**b**) 1× to 0.02× the reference K_fm_; (**c**) 0.01× to 0.001× the reference K_fm_; (**d**) the corresponding initial rate and the EC_50_ are shown as a function of the K_fm_, where the solid lines are not curve fittings but just a guide to the eye.

The results obtained by varying the formation equilibrium constant of the complex in the membrane K_fm_ are less straightforward and are shown in Figure 6.

It is quite counterintuitive to see in Figure 6a that transport efficiency decreases with increasing K_fm_, which means by increasing the anion-binding affinity of the carrier. Figure 6b shows that, in this specific condition (i.e., given the value of all the other parameters) it is a decrease in K_fm_ that produces an improvement of the transport. The maximum was reached with K_fm_ = 0.2 × K_fm,ref_. Below this threshold (Figure 6c), transport efficiency was found to progressively decrease with further decreasing levels of K_fm_. This trend is due to the interrelationship between K_fm_ and P. Of course, guest-binding affinity has to be large enough to have a significant amount of membrane-bound complex and, thus, a sufficient concentration gradient of the latter to drive its net flux towards the EV solution. However, a too large K_fm_ together with a large *p* value means that the final equilibrium will be less shifted towards the release of the guest, but towards the membrane-bound complex instead, thus, being detrimental for transport efficiency. Figure 6d clearly shows that K_fm_ should be neither too small nor too large and a wide acceptable range exists in between.

This is only in partial agreement with previous QSAR conclusions [23], on which bases a simple positive correlation was put forward between transport efficiency and anion-binding affinity. Another important observation is that significant transport activity is predicted for as small as 0.001 × K_fm,ref_. Such low affinity cannot be measured by ^1^H-NMR titration in solution and, thus, our results can explain how carriers with no detectable binding affinity for nitrate in DMSO-d_6_ solution can effectively transport this anion across phospholipid membranes.

**Figure 7:**
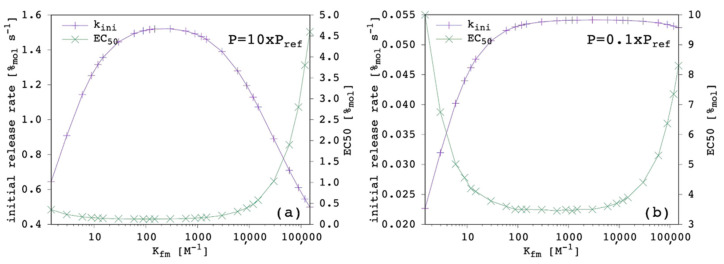
Simulated chloride transport according to the reference parameter set (Appendix A) by changing the complex formation equilibrium constant in the membrane: (**a**) P = 10 × P_ref_; (**b**) P = 0.1 × P_ref_. The initial rate and the EC_50_ are shown as a function of the K_fm_, where the solid lines are not curve fittings but just a guide to the eye.

Most importantly, whether increasing K_fm_ is an advantage is strictly case dependent. Figure 7 shows the results obtained when P was either increased or decreased by one order of magnitude.

When P was increased, k_ini_ was larger and EC_50_ was correspondingly smaller, as expected (Section 3.2). The k_ini_ appeared to be more sensitive to K_fm_ variation, i.e., the optimal range was narrower. In addition, the center of this optimal range was shifted to a lower value. The EC_50_ showed to be less sensitive on the low-end side of Figure 7a, which indicates that a larger lipophilicity allows the carrier to be a good one, even with a lower chloride affinity.

Conversely, when P was decreased, k_ini_ was smaller and EC_50_ was correspondingly larger, as expected (Section 3.2). The k_ini_ appeared to be less sensitive to K_fm_ variation when the latter was large, simply because the transport efficiency was poor, no matter how large the chloride affinity was. The EC_50_ was more sensitive on the low-end side of Figure 7b, which indicates that a reduced lipophilicity needs to be compensated by a large chloride affinity for the carrier to be effective.

Overall, our results show that P had the largest weight on transport efficiency, although K_fm_ was important too and the optimum was obtained through a balance between K_fm_ and P, in agreement with previous investigations [23,28]. We conclude that potential candidates with relatively low K_fm_ should not be discarded during first screenings. They might be good transporters, provided that P is large enough, as can be seen in the data reported in the literature [9,23,27,50]. Finally, for the sake of completeness, we found that K_fm_ affected the curvature of release curves more than P did, according to the present model.

### 3.4. Carrier Diffusion Coefficient through the Membrane

**Figure 8:**
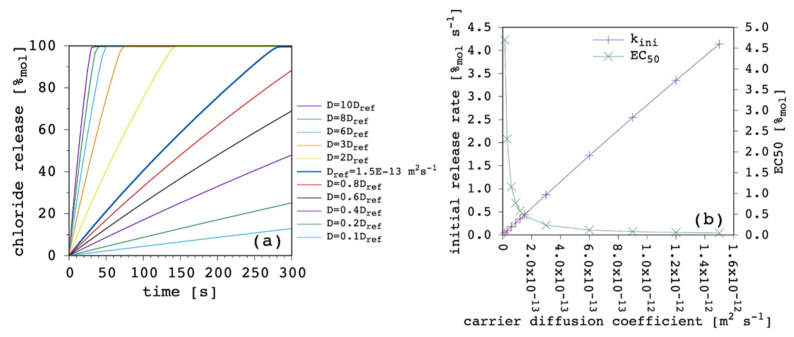
Simulated chloride transport according to the reference parameter set (Appendix A) by changing the carrier diffusion coefficient through the membrane: (**a**) release curves; (**b**) the corresponding initial rate and the EC_50_ are shown as a function of the carrier diffusion coefficient. The lines are not curve fittings but just a guide to the eye.

The third fundamental parameter is the carrier diffusion coefficient, D. Figure 8 shows the results obtained by varying the latter while keeping all the other parameters fixed.

The weight of this parameter over the transport process was huge. By changing only one order of magnitude we moved from extremely efficient to dramatically inefficient. Since the free carrier in the EV solution works as a reservoir (Section 3.1), its ability to cross the lipid bilayer, entering the IV solution and restoring the amount of the carrier that is leaving the vesicles in the form of complex is crucial to the overall transport rate. It is interesting to note how the curvature of release curves was not affected at all by D variations. In fact, k_ini_ was linearly dependent from D. The EC_50_ showed great changes when D was lower than the complex diffusion coefficient, but it tended to a plateau when it was larger. The D dominated transport efficiency, but no matter how fast new free carrier entered the vesicles, the diffusion of the complex became the limiting factor of the overall transport rate. For the sake of completeness, we also performed the same simulations with a larger value of the complex diffusion coefficient (6 × 10^−13^ m^2^ s^−1^) but the curves obtained were almost identical to those shown in Figure 8a,b.

### 3.5. Complex Diffusion Coefficient through the Membrane

**Figure 9:**
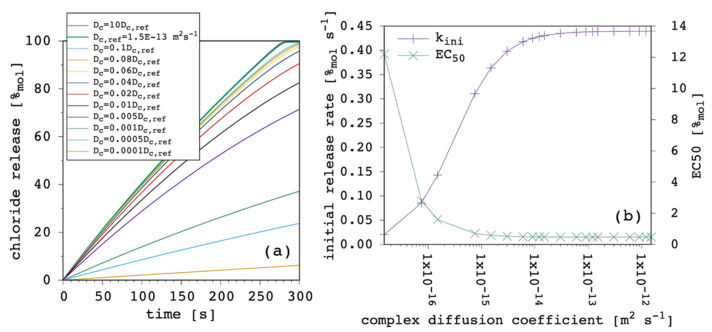
Simulated chloride transport according to the reference parameter set (Appendix A) by changing the complex diffusion coefficient through the membrane: (**a**) release curves; (**b**) the corresponding initial rate and the EC_50_ are shown as a function of the complex diffusion coefficient. The lines are not curve fittings but just a guide to the eye.

Our model has the interesting feature of taking the diffusion coefficient of the free carrier and the complex as explicitly distinct, so that it is possible to investigate their individual weight over the transport process. Figure 9 shows the results obtained by varying the complex diffusion coefficient, D_c_, while keeping all the other parameters fixed.

Even if D_c_ was larger than D, the latter dominated the transport process (Section 3.4). The weight of D_c_ could be seen only when it is significantly smaller than D, so that it became predominant as the rate limiting factor of the overall transport process. Although the free carrier was characterized by large diffusivity through the membrane, if the complex was not, the latter rapidly accumulated in the IV leaflet of the lipid bilayer and also inhibited the flux of the free carrier coming from the EV solution. We believe that this situation is not so uncommon in real cases, since it is not only the size that affects the diffusion coefficient [23], but also the net charge or, more generally, the charge distribution [42,43,44]. For example, in the case we took as the benchmark [22] (see the Methods), the carrier was uncharged while the chloride complex was negatively charged with a similar size, so it was plausible to expect that D_c_ < D.

**Figure 10:**
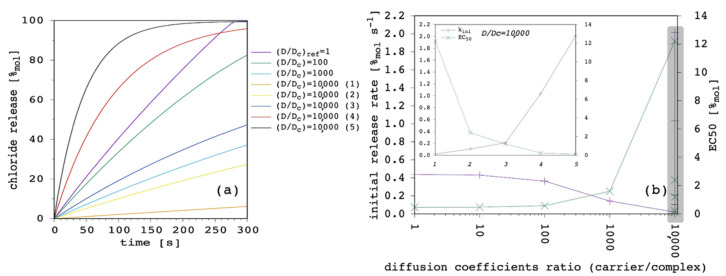
Simulated chloride transport according to the reference parameter set (Appendix A) by changing both the carrier and the complex diffusion coefficient through the membrane: (**a**) release curves; (**b**) the corresponding initial rate and the EC_50_ are shown as a function of the carrier to complex diffusion coefficient ratio. The lines are not curve fittings but just a guide to the eye. The inset is the expansion of the gray-shaded region of the plot, where the five simulations with D/D_c_ = 10,000 and labeled from 1 to 5 are shown from the left to the right, respectively.

At this point of the discussion, it is interesting to note how D_c_, not D, is able to affect the curvature of the release curves. The impact, of course, depends on the ratio between the two diffusion coefficients, as is shown in Figure 10.

Starting from D = D_c_ = 1.5 × 10^−13^ m^2^ s^−1^ (Appendix A), we performed a first series of simulations by reducing D_c_ by one order of magnitude in each case, in order to progressively increase the D/D_c_ ratio from 1 to 10,000. Then, we kept the latter fixed and we performed another series of simulations by increasing both D and D_c_ by a half order of magnitude in each case. These are labelled from (1) to (5) in Figure 10.

As far as the first series of simulations was concerned, it was clear that by reducing D_c_ but keeping D constant (and larger) transport efficiency progressively reduced until it was eventually negligible. The second series was even more interesting. Although the ratio between D and D_c_ was remarkably large, it was possible to move from extremely inefficient (dataset 10,000 (1) in Figure 10) to quite efficient transport (dataset 10,000 (5) in Figure 10). These simulations show that, according to the present model, D/D_c_ is extremely important to determine efficacy of the transport system, but also the absolute value of the two diffusion coefficients are fundamental and can make a great difference. In particular, D is the dominant parameter over k_ini_, while a smaller and smaller D_c_ increases the curvature of the release curve, thus, affecting the release at longer time.

Release curves reported in the literature are not straight lines up to 100%_mol_, but typically show a marked curvature, especially at large carrier concentrations [9,28,51]. Thus, we selected the case with D/D_c_ = 10,000 (4) reported in Figure 10 to perform further simulations.

### 3.6. High Carrier to Complex Diffusion Coefficient Ratio

As motivated in the previous section, we set the diffusion coefficient of the carrier through the membrane D = 7.5 × 10^−12^ m^2^ s^−1^, and that of the complex D_c_ = 7.5 × 10^−16^ m^2^ s^−1^, in order to have a ratio of 10,000 and a marked curvature of the release curve (red curve in Figure 10).

**Figure 11:**
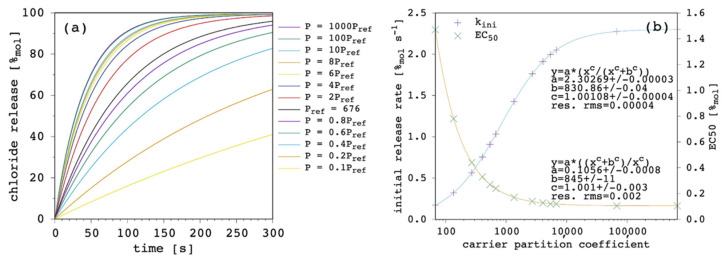
Simulated chloride transport according to the reference parameter set (Appendix A; D = 7.5 × 10^−12^ m^2^ s^−1^; D_c_ = 7.5 × 10^−16^ m^2^ s^−1^) by changing the carrier’s partition coefficient: (**a**) release curves; (**b**) the corresponding initial rate and the EC_50_ are shown as a function of the carrier’s partition coefficient. The least-square curve fittings are also shown.

First, we investigated the variation of the free carrier partition coefficient, P, as shown in Figure 11.

Despite the high D/D_c_ ratio and the curvature induced to the release curves, when Figure 11 is compared with Figure 5 (Section 3.2), it is clear that P affected the overall transport process in the same way. The k_ini_ increased according to a hyperbolic curve as a function of P, and EC_50_ decreased according to a reciprocal hyperbolic curve. We motivated this behavior by the increase in the amount of the membrane-bound species with increasing P and the consequent increase in its net flux, while the diffusion through the membrane eventually became rate limiting. The simulations presented in this section bolster that conclusion. In fact, the inspection of the curve fittings reported in Figure 11b and the comparison with Figure 5b reveal that values of k_ini_ were larger in the last simulations (and EC_50_ values were smaller), which was basically due to the larger D value applied (7.5 × 10^−12^ vs. 1.5 × 10^−13^ m^2^ s^−1^). Most importantly, k_ini_ reached the final plateau at a lower P, as demonstrated by the lower b parameters in the curve fittings equations, representing the *p* value at which half-maximum k_ini_ was obtained. This was clearly due to the lower D_c_ value applied in the last simulations (7.5 × 10^−16^ vs. 1.5 × 10^−13^ m^2^ s^−1^), thus, being already rate limiting over the entire transport process at lower *p* values.

**Figure 12:**
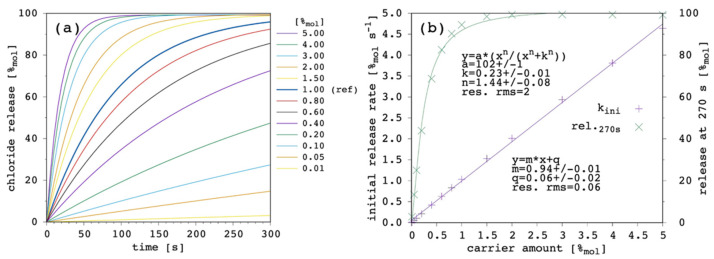
Simulated chloride transport according to the reference parameter set (Appendix A; D = 7.5 × 10^−12^ m^2^ s^−1^; D_c_ = 7.5 × 10^−16^ m^2^ s^−1^) by changing the amount of carrier: (**a**) release curves; (**b**) the corresponding initial rate and the release at 270 s are shown as a function of the carrier amount. The least-square linear curve fittings are also shown.

Figure 12 shows the results obtained as a function of the total carrier amount.

When these results were compared with Figure 3 (Section 3.1), given the larger value of D applied in the last simulations, k_ini_ values were larger and, of course, EC_50_ values were smaller. While k_ini_ still showed linear dependence on carrier amount, EC_50_ lacked the linearity we obtained when D = D_c_, and this was the most interesting result from these last simulations. Indeed, EC_50_ data now appear more compatible with the trends shown in the literature [23], showing a sigmoidal dependence on carrier amount (n parameter of the curve fitting > 1). This is due to the marked curvature acquired by the release curves, especially at a large carrier amount, which is ultimately due to the fact that D_c_ ≪ D.

### 3.7. Carrier Aggregation in Solution

**Figure 13:**
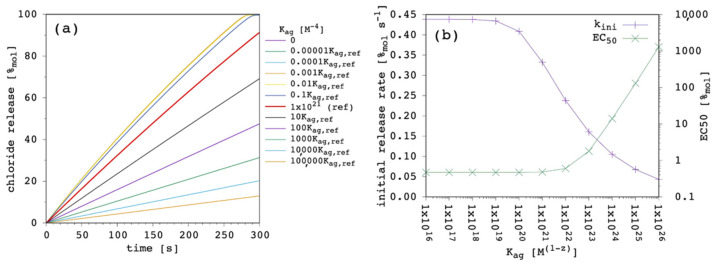
Simulated chloride transport according to the reference parameter set (Appendix A; aggregation number z = 5) by changing the aggregation equilibrium constant of the carrier in solution: (**a**) release curves; (**b**) the corresponding initial rate and the EC_50_ are shown as a function of the aggregation equilibrium constant. The lines are not curve fittings but just a guide to the eye.

Our present model is in agreement with the literature, showing that the carrier partition coefficient is one of the most important parameters affecting the transport performance [9,23,43,48]. At the end of Section 3.2, we concluded that, in some instances, increasing the carrier lipophilicity might provide only moderate advantages, while the risk of aggregation in solution becomes progressively more important. For this reason, we included reversible carrier aggregation in solution to open the possibility of taking this into explicit consideration when analyzing experimental data. Figure 13 shows the effect of changing the aggregation equilibrium constant, K_ag_.

The specific reference values of 1 × 10^21^ M^−4^ and 5 for the K_ag_ and aggregation number, respectively, were selected to have 25%_mol_ of the carrier in the aggregated form at the beginning of the simulation (red curve in Figure 13a). The results showed, as expected, that by increasing the aggregation of the carrier in solution, lesser and lesser carrier was available for transport, causing the k_ini_ to decrease. On the other hand, by decreasing the K_ag_ below a certain threshold, the transport efficacy (and the release curves) became virtually identical to the one obtained in the absence of aggregation. Overall, in the present conditions, release curves appeared not to be very sensitive to changes of K_ag_, as the latter need to be changed by orders of magnitude to observe significant differences. However, the effect on the EC_50_ was huge, as it can be observed in Figure 13b.

**Figure 14:**
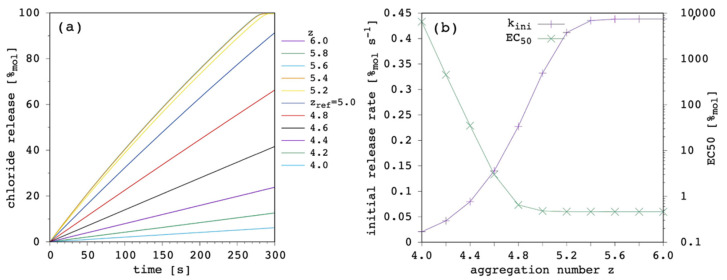
Simulated chloride transport according to the reference parameter set (Appendix A; K_ag_ = 1 × 10^21^ M^−4^) by changing the aggregation number of the carrier in solution: (**a**) release curves; (**b**) the corresponding initial rate and the EC_50_ are shown as a function of the aggregation number. The lines are not curve fittings but just a guide to the eye.

The effect of changing the aggregation number, z, was opposite with respect to the variation of K_ag_. This is due to the fact that at constant K_ag_, by increasing z, a larger number of monomers are needed to form a single aggregate. In turn, aggregation is less probable (or, a larger K_ag_ would be needed to reach the same number of monomers involved in aggregates). Aggregation decreases with increasing z, and above a certain threshold the transport efficacy (and the release curves) become virtually identical to the one obtained in the absence of aggregation (Figure 14a). Again, the effect on the EC_50_ is huge, as it can be observed in Figure 14b. When the effect of changing K_ag_ and z are compared, it appears that the release curve is much more sensitive to z variations.

The opposite dependence of transport efficacy on K_ag_ and z poses a serious limitation when this model is used for the curve fitting of experimental release data. In fact, such opposite dependance means that the same release curve can be obtained by many different combinations of K_ag_ and z values. For instance, we obtained almost perfectly superimposed curves with the following three combinations of K_ag_, z: (i) 1 × 10^22^, 5.2; 1 × 10^21^, 5.0; 1 × 10^20^, 4.8. This means that one of the two parameters, at least, should be experimentally determined. However, the value of K_ag_ basically depends on z. Determination of the aggregation number is tricky as far as we know, with techniques that have been specifically developed for micelles [52,53] and that possibly need to be adapted to the specific carrier of interest. In conclusion, although we opted for a rigorous microscopical description of the aggregation equilibrium in this first version of our model, aiming at capturing the potential impact on the transport process, we plan to move to a more empirical and macroscopical description in future versions.

**Figure 15:**
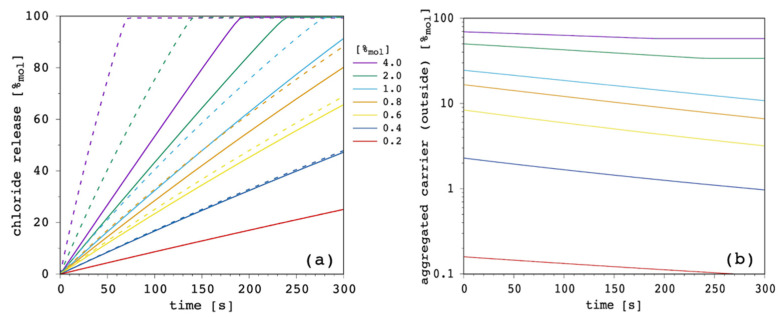
Simulated chloride transport according to the reference parameter set (Appendix A; K_ag_ = 1 × 10^21^ M^−4^; z = 5) by changing the amount of carrier: (**a**) release curves (solid lines) together with the corresponding results in the absence of aggregation (dashed lines); (**b**) amount of carrier in the aggregated form (not available for transport) in the EV solution as a function of time.

Finally, Figure 15 shows the effect of aggregation as a function of carrier total amount.

When the release curves with and without aggregation are compared (Figure 15a), we can clearly see that the weight of aggregation is very important at large carrier concentrations, while this becomes progressively negligible with decreasing amounts of the latter. In addition, Figure 15b shows that the amount of unavailable carrier in the form of aggregates decreases as a function of time, since the carrier total concentration in the EV solution slightly decreases along the simulation (Section 3.1).

### 3.8. Vesicles Curvature

In the first version of our model, for the sake of simplicity we decided to neglect vesicles curvature by using the same radius for both the outer and the inner surface. However, in order to assess the weight of the outer/inner radius difference, which is also related to the lipid bilayer thickness, we wrote a successive version to include this (see the Methods), in agreement with other models from the literature [20]. By taking different values for the outer and inner radii, different values for both the outer and inner area as well as for the inner volume were found, with respect to the first version of the model.

**Figure 16:**
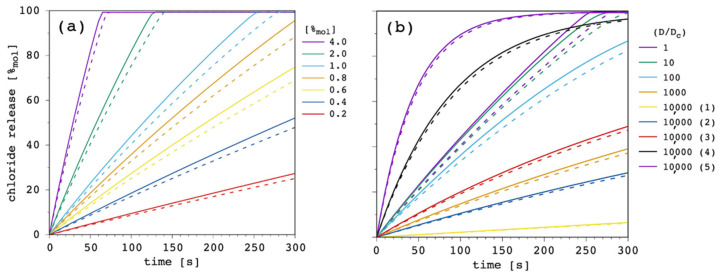
Simulated chloride transport according to the reference parameter set (Appendix A). Release curves obtained with (solid lines) and without (dashed lines) vesicles outer/inner different radius are shown: (**a**) by changing the amount of carrier; (**b**) by changing both the carrier and the complex diffusion coefficient through the membrane (same ratios as in Figure 10).

For any given carrier concentration, we found better transport with almost constant difference from the first version of the model up to the final equilibrium (Figure 16a). This is due to the inner volume contraction, which generates larger concentrations and, thus, larger outgoing flux for the same amount of incoming moles. However, if a larger and larger ratio between the carrier and the complex diffusion coefficient through the membrane was considered (Figure 16b), the weight of a different outer/inner radius became negligible in the present conditions.

Nevertheless, by including this detail we made the model more general—as we cannot rule out its importance when lipids are changed—to vary the membrane thickness, and/or consider when the average size of the vesicles is changed.

### 3.9. Curve Fitting of Experimental Data

In the present version of the algorithm, the curve-fitting of each single experimental dataset is individually performed. The only fitting parameters are the two diffusion coefficients through the membrane, D and D_c_. All the other parameters are specified as input to the code, as provided by experiments or predicted from established informatic tools (see the Methods).

**Figure 17:**
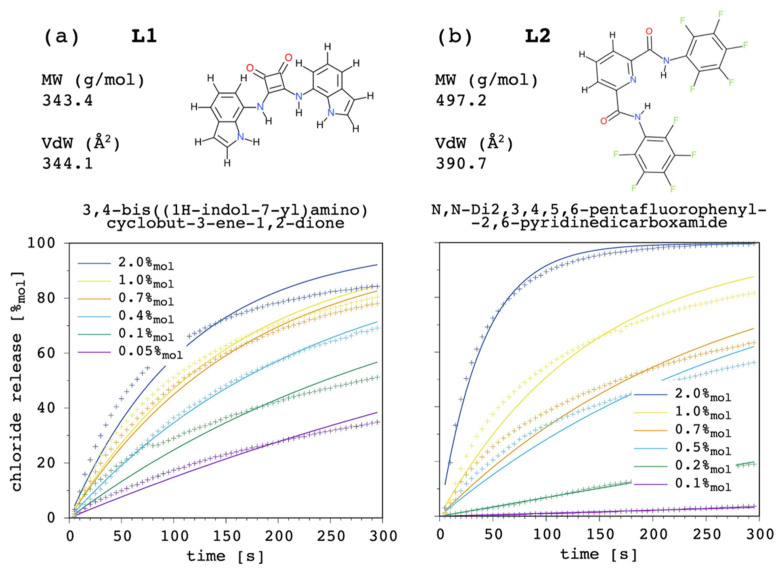
Curve-fitting (solid lines) of experimental chloride transport data (points) for two carriers of different type at different concentrations: (**a**) L1, 3,4-bis((1H-indol-7-yl)amino)cyclobut-3-ene-1,2-dione), and (**b**) L2, N,N-Di2,3,4,5,6-pentafluorophenyl-2,6-pyridinedicarboxamide. Molecular weight (MW) was calculated with Avogadro [56,57]. The Van der Waals surface (VdW) was calculated with PyMOL [58].

As examples, we selected the above-mentioned squaramide [22] (3,4-bis((1H-indol-7-yl)amino)cyclobut-3-ene-1,2-dione), L1, with $P=6.76\;\times \;10^{2}$ and $K_{\mathrm{fm}}=1.48\;\times \;10^{3}\;M^{-1}$, and a recently reported [54,55] dipicolineamide (N,N-Di2,3,4,5,6-pentafluorophenyl-2,6-pyridinedicarboxamide), L2, with $P=1{.62\times 10}^{4}$ and $K_{\mathrm{fm}}=4{.70\;\times \;10}^{1}{\;M}^{-1}$. These two chloride carriers were selected because of their different P as well as K_fm_ value. Namely, L2 has a larger membrane affinity (PL2PL1=24), whereas L1 has a larger chloride affinity (Kfm,L1Kfm,L2=31). In both cases, carrier aggregation in solution was switched off by setting K_ag_ = 0. Figure 17 shows the results.

Although the present model can certainly be improved (see Section 3.10), and the experimental determination of the input parameters could be obtained with more accurate approaches, curve fittings were rather satisfactory overall, showing how the present simulation model can be applied to quite different systems. The results bolster what has been emphasized throughout the discussion. If we compared the curves at 1.0%_mol_ only, a comparable transport efficacy would be deduced for these two carriers. Although L1 does not have a dramatic affinity for the lipid bilayer, its four NH donors provide the carrier with a very good chloride affinity. On the other hand, although L2 does not have a remarkable affinity for the guest, perfluorination of the pendants was successful in providing the carrier with a very good membrane affinity. However, the analysis cannot rely on one single curve, as these fundamental differences appear reflected by the release curves of L1 being far less affected by changes in the carrier amount, when compared to those obtained for L2 (EC_50_ was experimentally estimated as 0.11%_mol_ with respect to total lipid, or 0.55 μM, [22] and 0.44%_mol_, or 2.20 μM [54], respectively). In addition, the weight of possibly different diffusion coefficients through the membrane cannot be ruled out.

Our curve fittings showed that, on average, the diffusion coefficient of the free carrier was 2 × 10^−10^ and 1 × 10^−11^ m^2^ s^−1^ for L1 and L2, respectively. This difference correlates with L2 having both molecular mass and a Van der Waals surface larger than L1 (Figure 17). The diffusion coefficient of the complex was found to be several orders of magnitude lower than that of the corresponding free carrier, as expected. In particular, D/D_c_ was found to be ca. 3 × 10^5^ and 3 × 10^3^ for L1 and L2, respectively. Then, on the contrary, with respect to the free carrier, the diffusion coefficient of the complex formed by L1 was the smallest, with value of 7 × 10^−16^ and 3 × 10^−15^ m^2^ s^−1^ for L1 and L2, respectively.

Such a big difference in D_c_ falling among L1 and L2 cannot be correlated to the different size of the complex. It is plausible to hypothesize that the weight of charge distribution and polarizability, in addition to polar/unpolar surface ratio and conformational flexibility of the complex, has been underrated in the literature. Even the simplest transport mechanism modelled here appears to be highly multi-variate, with a deep interplay among the parameters, which is hard to fully understand without proper and detailed models and simulation methods.

### 3.10. Technical Remarks

The introduction of the reversible aggregation of the carrier in solution led us to move from the analytical to the numerical solution of the equation needed to calculate the concentration of the free carrier in solution at each time-step. This modification caused a dramatic reduction in the algorithm performance. Just to give an idea, on an AMD Opteron 6174 with 2.2 GHz clockspeed, it takes about 20–30 s to simulate 300 s at 1 × 10^−6^ s time-step with the analytical solution. With the numerical approach, the same simulation takes as long as ca. 15 min. This is not a problem if one only wants to simulate and compare the curves for different values of the parameters, but if the goal is the curve fitting of experimental data, with our Monte Carlo approach, the same simulation has to be performed from hundreds to few thousands of times. With the analytical solution, the curve fitting of one single dataset was accomplished in about 6 h. With the numerical approach, the same goal was achieved in 10 days, which is clearly unacceptable.

Thus, we moved from the fixed to the variable time-step approach, which practically means that every 5 s of simulated time (i.e., the dwell time of experimental data), fluxes are checked, and the time-step is possibly increased (see the Methods). This approach proved to generate virtually identical output with enormous gain in performance. We basically moved back to the speed obtained with the analytical solution or even faster, such that the curve-fitting of one single experimental dataset took a few hours.

This goal was a milestone to us, since now the algorithm is not limited to very simple models whose equations are analytically solvable, but it can be extended to more complex scenarios. We are going to include the charge gradients, which are completely neglected at the moment, meaning that we will also be able to simulate electrogenic transports. We are also going to take the counter-diffusing ion into explicit account with its own permeability. The pH and the buffer need to be explicitly included in order to open our approach to protonophores.

## 4. Conclusions

The presented model (and the algorithm) for the carrier-assisted transport of chloride across lipid membranes allow a detailed study of the impact of several important parameters on the transport efficiency of the anionophore. Despite its apparent simplicity, the present method was able to confirm, and sometimes to extend, the current comprehension of the complicated interplay of the parameters accounting for the uniport mechanism. Besides the strong role of the carrier’s partition into the membrane, the interplay with the complex formation equilibrium constant showed the optimal range for the latter, including potentially good transporters with low anion affinity. In addition, the weight of the diffusivity of the carrier and its complex through the membrane were treated separately, and differently from other methods reported in the literature. We think that the present approach also gives satisfactory results in terms of the experimental data fitting and, therefore, it is worthy of being developed, improved, and extended to cover more complex and general scenarios, including the exploration of anion transport selectivity. Likewise, the experimental determination of the parameters can certainly be improved and, although we have applied experimental determinations typical in the field, new methods or more accurate determinations might certainly be involved in the future. Overall, the presented strategy represents a potent tool to unveil important quantitative details of the anion transport mechanisms across lipid membranes by artificial receptors that can only be monitored by means of detailed numerical simulations. These hints could be most useful to guide the design of and to identify new highly efficient anion carriers.

## Data Availability

The data presented in this study are available on request from the corresponding author. New datasets with different values for the parameters and experimental data curve-fitting can be provided upon request by the corresponding author. The algorithm will be made available through a public repository as soon as the planned developments and extensions will be included by the authors.

## Appendix A

**Table A1:** Input parameters used to generate the reference data for all the numerical tests reported in the manuscript.

Description	Unit	Value
Total lipid concentration	${\mathrm{mol}\;L}^{-1}$	${5\;\times \;10}^{-4}$
Total solution volume	L	${5\;\times \;10}^{-3}$
Average vesicles diameter	m	${2\;\times \;10}^{-7}$
(Hydrophobic) Thickness of the lipid bilayer	m	$2{.85\;\times \;10}^{-9}$
Lipid surface density	$m^{-2}$	$1{.3\;\times \;10}^{18}$
Starting amount of carrier in the external solution (with respect to total lipid)	${\%}_{\mathrm{mol}}$	$1.0{\;\times \;10}^{0}$
Starting amount of carrier in the internal solution	mol	0.0
Starting guest concentration in the internal solution	${\mathrm{mol}\;L}^{-1}$	$4{.89\;\times \;10}^{-1}$
Starting guest concentration in the external solution	${\mathrm{mol}\;L}^{-1}$	0.0
Carrier membrane/water partition coefficient		$6{.76\;\times \;10}^{2}$
Complex formation equilibrium constant on membrane	${\left({\mathrm{mol}\;L}^{-1}\right)}^{-1}$	$1{.48\;\times \;10}^{3}$
Complex formation equilibrium constant in solution	${\left({\mathrm{mol}\;L}^{-1}\right)}^{-1}$	$8{.34\;\times \;10}^{-9}$
Carrier aggregation equilibrium constant (with aggregation number z)	${\left({\mathrm{mol}\;L}^{-1}\right)}^{-z+1}$	0.0
Carrier aggregation number		1.0
Lipid molar volume	${L\;\mathrm{mol}}^{-1}$	$7{.62\;\times \;10}^{-1}$
Membrane surface occupied by one carrier molecule	$m^{2}$	$1{.11\;\times \;10}^{-18}$
Membrane surface occupied by one complex molecule	$m^{2}$	$1{.11\;\times \;10}^{-18}$
Carrier diffusion coefficient through the membrane	$m^{2}s^{-1}$	$1{.5\;\times \;10}^{-13}$
Complex diffusion coefficient through the membrane	$m^{2}s^{-1}$	$1{.5\;\times \;10}^{-13}$
Simulation time	s	$2{.95\;\times \;10}^{2}$
Initial simulation time-step	s	$1{.0\;\times \;10}^{-6}$
Maximum simulation time-step	s	$1{.0\;\times \;10}^{-3}$
Output files writing frequency	step	$5{.0\;\times \;10}^{6}$
Maximum number of iterations (during curve-fitting)		$1{.0\;\times \;10}^{3}$
Experimental data recording dwell-time	s	$5{.0\;\times \;10}^{0}$
